# Non-Invasive Identification of Nutrient Components in Grain

**DOI:** 10.3390/molecules26113124

**Published:** 2021-05-24

**Authors:** Charles Farber, A. S. M. Faridul Islam, Endang M. Septiningsih, Michael J. Thomson, Dmitry Kurouski

**Affiliations:** 1 Department of Biochemistry and Biophysics, Texas A&M University, College Station, TX 77843, USA; farb1703@tamu.edu; 2 Department of Soil and Crop Sciences, Texas A&M University, College Station, TX 77843, USA; farid-bge@tamu.edu (A.S.M.F.I.); eseptiningsih@tamu.edu (E.M.S.); m.thomson@tamu.edu (M.J.T.); 3 The Institute for Quantum Science and Engineering, Texas A&M University, College Station, TX 77843, USA

**Keywords:** grain, *Oryza sativa*, nutrient content, Raman spectroscopy

## Abstract

Digital farming is a modern agricultural concept that aims to maximize the crop yield while simultaneously minimizing the environmental impact of farming. Successful implementation of digital farming requires development of sensors to detect and identify diseases and abiotic stresses in plants, as well as to probe the nutrient content of seeds and identify plant varieties. Experimental evidence of the suitability of Raman spectroscopy (RS) for confirmatory diagnostics of plant diseases was previously provided by our team and other research groups. In this study, we investigate the potential use of RS as a label-free, non-invasive and non-destructive analytical technique for the fast and accurate identification of nutrient components in the grains from 15 different rice genotypes. We demonstrate that spectroscopic analysis of intact rice seeds provides the accurate rice variety identification in ~86% of samples. These results suggest that RS can be used for fully automated, fast and accurate identification of seeds nutrient components.

## 1. Introduction

Digital agriculture is an emerging paradigm that aims to maximize crop yields while simultaneously minimizing the environmental impact of farming. Crop yields can be maximized by timely detection and identification of biotic stresses [1,2]. This information can be used for site- and dose-specific applications of fungicides and pesticides to cease the proliferation of pathogens and minimize crop losses associated with plant diseases. Abiotic stresses, including drought and nutrient deficiencies, can cause even higher crop losses than biotic stresses [3,4]. If detected early, water and fertilizers can be administrated to mitigate crop losses caused by these stresses. Digital farming also requires advanced methodologies for plant breeding and selection to develop a germplasm of crops that includes varieties with higher drought or salinity tolerance as well as enhanced resistance to pathogens. A major drawback of conventional phenotyping techniques is the long period of time required to measure the effect of stress on plants [5,6]. Since destructive, traditional wet lab methods take a long time to get the nutritional data of a germplasm or breeding population, RS could be a good alternative for screening the germplasm or breeding population quickly, helping the breeder in selection for crop improvement.

Raman spectroscopy (RS) is an analytical technique that can be used to detect plant biotic and abiotic stresses, as well as to identify plant varieties and probe their nutritional content [7,8,9]. For instance, Farber and co-workers demonstrated that RS could be used to identify whether maize kernels were healthy or infected by *Aspergillus flavus*, *A. niger*, *Fusarium* spp., or *Diplodia* spp. with 100% accuracy [10]. Recently, Sanchez and co-workers demonstrated high accuracy of diagnostics of Huanglongbing (HLB) or citrus greening on both orange and grapefruit trees using RS [11]. Moreover, the researchers demonstrated that spectra of HLB-infected plants are drastically different from plants that experienced nutritional deficiencies. These results suggested that RS can be used to distinguish between biotic and abiotic stresses. RS is a non-invasive, non-destructive and a chemical-free technique. Thus, the direct cost of analysis is small [2]. Over the last decade, several companies have developed hand-held spectrometers that could be used in field to probe plant health in real time [10,11,12]. This eliminates the need for transportation of plant material, further reducing the cost of analysis, simultaneously minimizing the potential spread of pathogens. Experimental results reported by the our group [10,11,13,14,15,16,17,18,19,20] and other research laboratories [12,21,22,23,24] show that hand-held Raman spectrometers could be used for fast (one second spectral acquisition time) and nearly 100% accurate diagnostics of biotic and abiotic stresses [10,11,13,16,19,20,25]. This innovative sensing approach is based on detecting and identifying pathogen-induced changes in plant biochemistry. RS can be used to distinguish between biotic and abiotic stresses [11], identify pathogens on the species level [10] and reveal the cause of abiotic stresses [26]. Our group has shown that RS can be used for non-invasive plant phenotyping [14,15,27]. We also found that RS can differentiate between nematode-resistant and nematode-susceptible peanut varieties [15]. Our recent findings show that RS can be used to identify varieties of plant species based on the unique spectroscopic signatures of their leaves and seeds [15,27,28]. Moreover, RS enables non-invasive and non-destructive assessment of the nutrient content of seeds, that is, carbohydrate, protein, fiber, oil, and unsaturated fatty acids in peanut seeds [15,27].

The question to ask is whether RS can identify the nutritional content of the intact grain. To answer this question, we collected Raman spectra from intact seeds of 15 different rice varieties. We employed chemometric tools to identify and differentiate these varieties. Our findings suggest that Raman spectra acquired from the intact rice grain are dominated by vibrational bands originating from starch and fiber. We performed detailed spectroscopic analysis of these compounds to confirm these hypotheses. Finally, we investigated the extent to which RS can be used to determine the nutritional content of intact corn kernels in the corn cob. Our results suggest that more sophisticated spectroscopic approaches are required to deliver the laser light through opaque and light-scattering husk material. This obstacle can be partially overcome by spatially offset Raman spectroscopy (SORS). Our results show that although SORS is not capable of probing the composition of intact corn kernels through the intact husk, decent signal-to-noise spectra can be obtained through two layers of husk material with 3–4 mm offset. These results further reflect the potential of RS in assessment of the nutritional content of intact seeds and spectroscopic identification of rice varieties.

## 2. Results and Discussion

**Figure 1:**
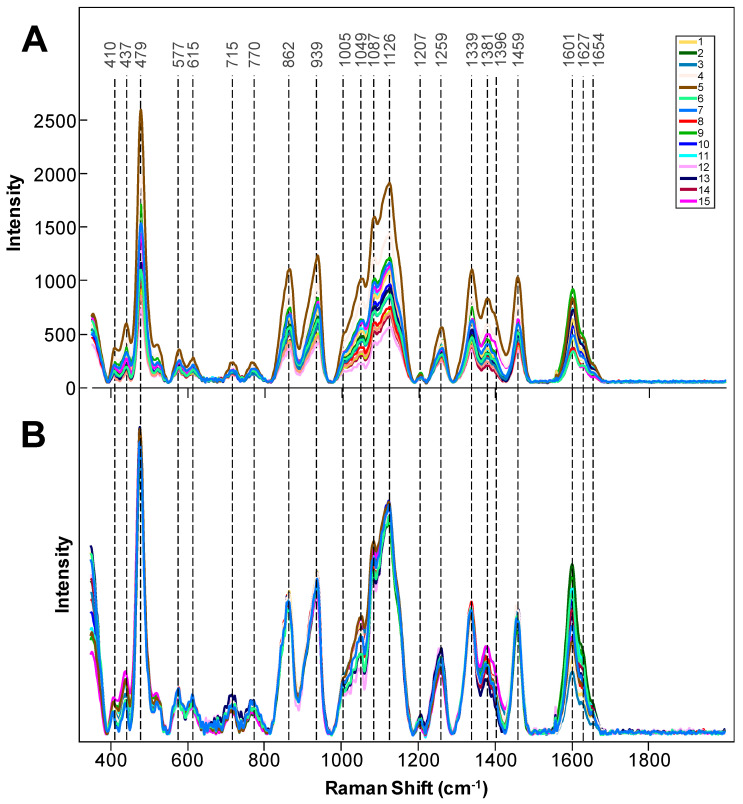
Raw (**A**) and area normalized (**B**) Raman spectra collected from intact seeds of 15 different rice genotypes.

Raman spectra collected from intact rice seeds exhibit vibrational bands that can be assigned to carbohydrates (410–1259 cm^−1^), aliphatic (CH_2_ and CH_2_/CH_3_ vibrations) (1339–1459 cm^−1^), and lignin (1601–1633 cm^−1^) (Table 1 and Figure 1). The vibrational band at 1005 cm^−1^ can be assigned to both carotenoids and proteins. Therefore, this band cannot be used for unambiguous interpretation of the nutritional content of rice seeds and will be excluded from the band analysis. These findings suggest that spectroscopic analysis of interact seeds can be used to probe the content of starch (410–1259 cm^−1^) and fiber (1601–1633 cm^−1^). 

**Figure 2:**
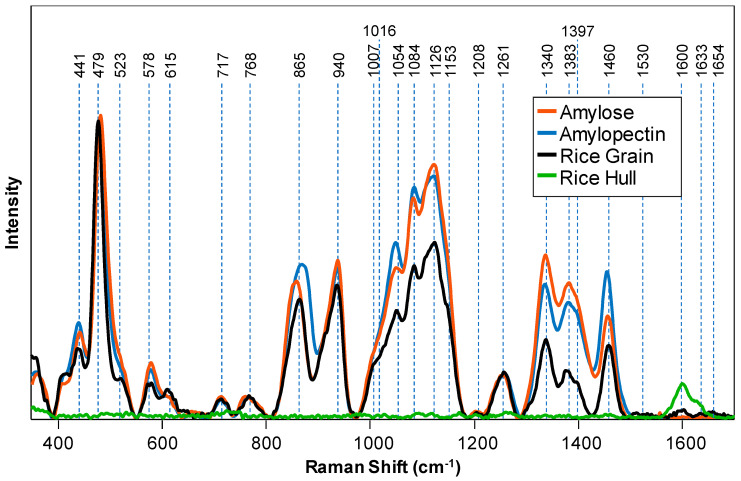
Raman spectra of amylose, amylopectin, intact rice grain and rice hull.

To prove the accuracy of the band assignment, we collected the spectra from amylose and amylopectin, two essential components of starch, as well as from the rice hull and de-hulled, cleaned rice (Figure 2). We found that amylose and amylopectin had very similar spectra. If normalized on the intensity of 479 cm^−1^, the spectrum of amylopectin had slightly more intense 865 cm^−1^, 1054 cm^−1^ and 1460 cm^−1^ bands, whereas the intensity of 1340, 1383 and 1397 cm^−1^ bands was found to be slightly lower than in the spectrum of amylose. This experimental evidence confirmed that the observed 410–1459 vibrational bands in the spectrum of the intact rice seeds originated from starch. Our findings also demonstrated that RS could not be used for differentiation between amylose vs. amylopectin in the grain. Thus, only total starch content could be probed in the intact rice using RS. 

We have also found that the spectrum of the rice hull is dominated by two vibrational bands at 1601–1630 cm^−1^ that originate from aromatic vibrations of polyphenols, the building blocks of lignin. This finding suggests that fiber content in the intact grain cannot be determined because this spectral region is obscured by the vibrations originating from the hull. Spectroscopic analysis of amylose, amylopectin, rice hulls and de-hulled rice revealed that only amount of the starch can be determined in the intact rice.

Next, we used OPLS-DA to determine the extent to which RS can be used for the quantitative identification of rice varieties based on their spectroscopic signatures. The loading plot and confusion matrix were then generated using this model, which contained nine predictive components (PCs) and 1087 (607–1693 cm^−1^) original wavenumbers from the standard normal variate (SNV) pre-processed first derivative spectra. The nine PCs explained a total of 33% variation between the classes. The model identified the oils peak at 1440 cm^−1^, the protein peaks at 1000 cm^−1^ (PC1) and 1655 cm^−1^ (PC2), the fiber peak at 1601 cm^−1^ (PC2) as the strongest predictors of rice variety, which supports the conclusions of our qualitative spectral analysis discussed above. The model also explained 55% of the variation (R2X) in the spectra. 

Our results demonstrated that RS can be used for highly accurate (86.2%) identification of rice varieties through intact rice hulls (Table 2). This high accuracy can be explained by the very low if any metabolic rates in the seeds as compared to actively growing plants.

**Figure 3:**
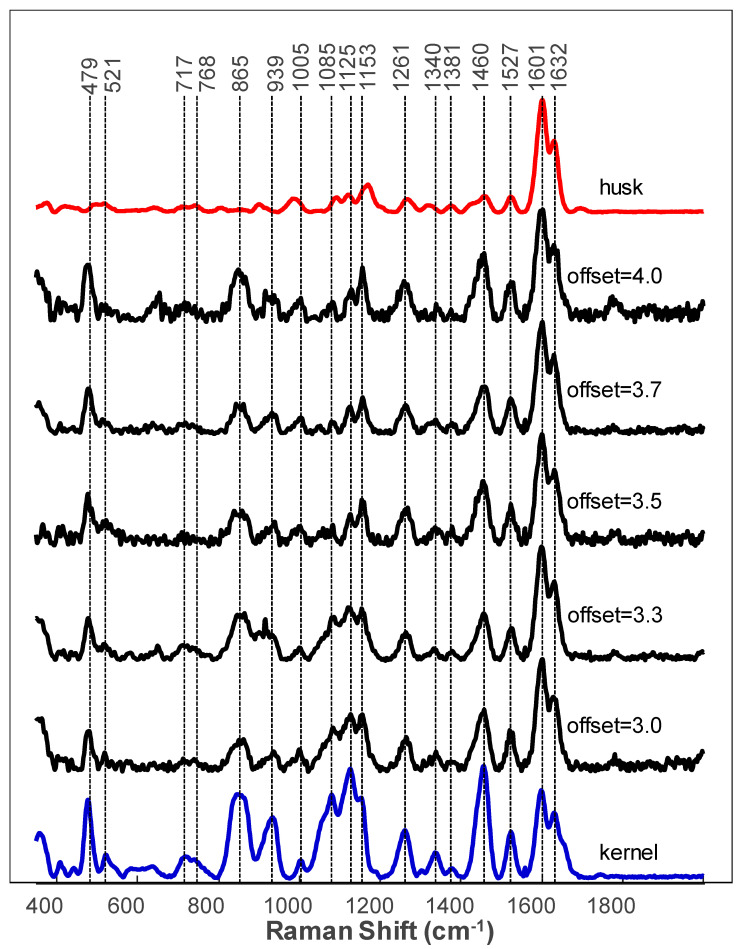
Raman spectra of corn husk (black) and kernels (maroon), as well as offset Raman spectra collected from the corn cob with two layers of husk material.

Our group previously demonstrated that RS could be used for highly accurate identification of corn kernels and nutritional assessment of corn starch, fiber, protein and carotenoids content [27]. The accuracy of these analyses was confirmed by Dumas Combustion Analysis, Megazyme Assay and Near-Infrared (NIR) readings form the same corn varieties. In this study, we investigated the extent to which RS could be used to access the nutritional values of the intact corn kernels in the corn cob. We found that corn husk both blocked and dispersed laser light preventing acquisition of spectra from the intact corn kernels. Only vibrational bands that originated from phenolic compounds could be observed in such spectra (Figure 3). We hypothesized that this problem could be overcome by spatially offset Raman spectroscopy (SORS). In SORS, two types of spectra are collected in a sequential order. The first type of spectra was collected when both excitation and collection axes were spatially co-aligned. These were typical normal Raman spectra that were discussed above. The second type of spectra was collected with spatial offsets between the excitation and collection optical axes. These spectra were called ‘offset Raman spectra’. This spectral collection approach allowed for probing deeper layers of the analyzed sample. First pioneered by Matousek group, SORS quickly became broadly utilized to detect explosives and illicit drugs in opaque bags and containers [37,38,39]. There is a growing body of evidence suggesting that SORS can be used to probe brain biochemistry through the intact skull [40]. Our group previously demonstrated that SORS could be used to probe nutritional content of intact potato tubers though the opaque peal [41]. 

The use of SORS for analysis of the intact corn cob showed that SORS failed to read the content of intact corn kernels (data not shown). We found that only if the husk were partially peeled and two husk layers remained, SORS could reveal the vibrational signatures of corn kernels (Figure 3). It should be noted that excellent signals from the underlying corn kernels were obtained at all offsets ranging from 3.0 to 4.0 mm. In the acquired offset Raman spectra, we observed vibrational bands originating from carbohydrates and carotenoids. Similar to the discussed above rice hull, we found that corn husk provides strong signals of the fiber (1601–1632 cm^−1^). These signals obscure readings of the fiber content of the intact corn kernels. 

## 3. Materials and Methods

### 3.1. Raman Spectroscopy 

**Figure 4:**
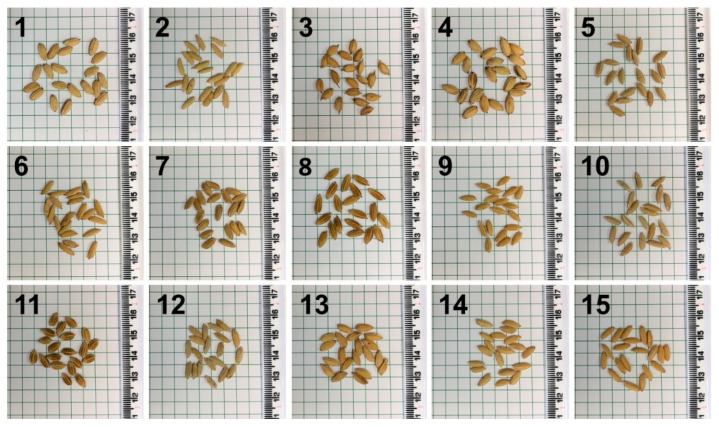
Photographs of seeds of 15 different rice varieties.

Spectra from intact seeds of 15 different rice varieties (Figure 4) were collected using a hand-held Resolve Agilent spectrometer (Agilent Technologies, Santa Clara, CA, USA) equipped with 830 nm laser. The following experimental parameters were used for all collected spectra: 1 s acquisition time, 1 accumulation, 495 mW power, surface scanning mode, and baseline spectral subtraction by device software. Spectra from the corn cob were acquired with the same settings as described for rice except that the instrument was in through-barrier mode, the offset distance was changed from 0–5 mm, and 10 accumulations were used instead of 1. 

### 3.2. Statistical Analysis

Orthogonal partial least-squares discriminant analysis (OPLS-DA): all collected Raman spectra were imported into SIMCA 14 (Umetrics, Umea, Sweden) for statistical analysis and scaled to unit variance to give all spectral regions equal importance. OPLS-DA was performed to determine the number of predicting and orthogonal significant components and identify spectral regions that best explain the separation between the classes. 

## 4. Conclusions

This work demonstrated that RS can be used for identification of the intact rice. OPLS-DA results show that rice seeds can be identified with on average 86.2% accuracy. This work also revealed limitations of RS and SORS for nutrient analysis. We found that a thick husk limited direct assessment of the nutritional content of corn kernels. This information could be revealed only if only two layers of husk material obscured the kernels. 

## Figures and Tables

**Table 1:** Vibrational bands and their assignments for spectra collected form leaves and seeds of peanuts.

Band	Vibrational Mode	Assignment
410–479	C-C-O and C-C-C deformations; related to glycosidic ring skeletal deformations δ(C-C-C) + τ(C-O) scissoring of C-C-C and out-of-plane bending of C-O	Carbohydrates [29]
577–615	ν(C-O-C) Glycosidic	Carbohydrates [29]
715–770	δ(C-C-O)	Carbohydrates [29]
862–937	(C6–C5–O5–C1–O1)	Carbohydrates [29]
1005	In-plane CH_3_ rocking of polyene aromatic ring of phenylalanine	Carotenoids [30]; protein
1049	ν(C-O) + ν(C-C) + δ(C-O-H)	Cellulose, lignin [31]
1087	ν(C-O) + ν(C-C) + δ(C-O-H)	Carbohydrates [29]
1126	ν(C-O) + ν(C-C) + δ(C-O-H)	Carbohydrates [29]
1207	δ(C-C-H)	Carbohydrates [29]
1259	Guaiacyl ring breathing, C-O stretching (aromatic); -C=C-	Lignin [32], carbohydrates, [29] unsaturated fatty acids [33]
1339	ν(C-O); δ(C-O-H)	Aliphatic, [34] carbohydrates [29]
1381–1396	δCH_2_ bending	Aliphatics [34]
1460	δ(CH_2_) + δ(CH_3_)	Aliphatics [34]
1601–1627	ν(C-C) aromatic ring + σ(CH)	Lignin [35,36]

**Table 2:** OPLS-DA confusion matrix of Raman spectra collected from seeds of 15 different varieties (1–15) of rice.

Genotype Number	Members	True Positive Rate	1	2	3	4	5	6	7	8	9	10	11	12	13	14	15
1	20	75%	15	0	1	4	0	0	0	0	0	0	0	0	0	0	0
2	21	81%	0	17	0	0	0	0	0	1	2	1	0	0	0	0	0
3	20	90%	1	0	18	0	0	0	0	0	0	1	0	0	0	0	0
4	13	85%	1	0	0	11	0	0	0	0	0	0	0	0	1	0	0
5	20	80%	0	0	3	0	16	0	0	0	0	1	0	0	0	0	0
6	21	76%	0	1	0	0	0	16	0	0	1	1	0	0	0	0	2
7	20	85%	0	0	0	0	0	0	17	0	0	2	0	1	0	0	0
8	20	85%	0	0	0	0	0	0	0	17	1	0	1	0	0	0	1
9	20	95%	0	1	0	0	0	0	0	0	19	0	0	0	0	0	0
10	20	90%	0	0	1	0	0	0	0	0	0	18	0	1	0	0	0
11	20	90%	0	0	0	0	0	0	0	0	2	0	18	0	0	0	0
12	16	87%	0	0	0	0	0	0	0	0	0	2	0	14	0	0	0
13	20	100%	0	0	0	0	0	0	0	0	0	0	0	0	20	0	0
14	20	75%	0	0	0	1	0	1	0	0	2	0	0	0	0	15	1
15	20	100%	0	0	0	0	0	0	0	0	0	0	0	0	0	0	20
Total	342	86.2%															

## Data Availability

Data are available on reasonable request to the corresponding author.

## Sample Availability

Samples of compounds are available on reasonable request to the corresponding author.
